# Statistical Modeling of Single Target Cell Encapsulation

**DOI:** 10.1371/journal.pone.0021580

**Published:** 2011-07-21

**Authors:** SangJun Moon, Elvan Ceyhan, Umut Atakan Gurkan, Utkan Demirci

**Affiliations:** 1 Demirci Bio-Acoustic-MEMS in Medicine (BAMM) Laboratory, Center for Bioengineering, Brigham and Women's Hospital, Harvard Medical School, Boston, Massachusetts, United States of America; 2 Department of Mathematics, Faculty of Sciences, Koç University, Istanbul, Turkey; 3 Harvard-MIT Division of Health Sciences and Technology, Massachusetts Institute of Technology, Cambridge, Massachusetts, United States of America; University of South Florida, United States of America

## Abstract

High throughput drop-on-demand systems for separation and encapsulation of individual target cells from heterogeneous mixtures of multiple cell types is an emerging method in biotechnology that has broad applications in tissue engineering and regenerative medicine, genomics, and cryobiology. However, cell encapsulation in droplets is a random process that is hard to control. Statistical models can provide an understanding of the underlying processes and estimation of the relevant parameters, and enable reliable and repeatable control over the encapsulation of cells in droplets during the isolation process with high confidence level. We have modeled and experimentally verified a microdroplet-based cell encapsulation process for various combinations of cell loading and target cell concentrations. Here, we explain theoretically and validate experimentally a model to isolate and pattern single target cells from heterogeneous mixtures without using complex peripheral systems.

## Introduction

Cell encapsulation in nanoliter volume droplets and patterning has a broad range of applications including tissue engineering using biodegradable hydrogels [1], cell printing [2], [3], [4], [5], [6], [7], [8], [9], cell sorting [10], cryobiology [3], [11], stem cell differentiation [12], [13], and single cell genomics [14]. These broad applications emphasize the need to develop and understand the dynamics of cell encapsulation and isolation processes. There are various technologies reported to encapsulate cells in media and hydrogel droplets [15], [16], [17]. The conventional inkjet printing systems were adapted [15] as tools to encapsulate cells in droplets and pattern these cell-encapsulating droplets [18]. There are microfluidic based cell encapsulation techniques [19], which offer limited control over the droplet and its position after encapsulation. Cell printing techniques employ fewer handling steps to pattern cells and allow handling of a few cells encapsulated in a single droplet at a time (i.e., drop-on-demand) compared to the existing large volume methods such as manual cell dilutions. Three inkjet based droplet generation mechanisms have been reported, i.e., thermal jet [20], bubble jet [21], and piezo-actuator based ejector [22]. These technologies face viability and functionality challenges in post-printing due to heat and shear effects on cells during droplet generation [18], [23], [24]. Recently, a laser assisted cell patterning system was developed for cell encapsulation and printing [25]. Alternative approaches such as single to few cells encapsulated in droplets by acoustic droplet generators were demonstrated [2], [4], and cell-laden hydrogel droplets were generated by a mechanical solenoid valve in a high-throughput manner [5] [7], [8], [9]. These approaches could alleviate shear forces to cells, since droplet volume is relatively larger than total volume of encapsulated cells and lead to high cellular viability and functionality. All of the methods listed above aim to precisely control cell density in encapsulated droplets. However, a statistical model that can effectively predict the target cell encapsulation phenomena from a heterogeneous population has not been developed.

Challenges still remain to enable efficient extraction, isolation, and patterning of cells from heterogeneous cell suspensions and to keep them alive throughout the process. Although microfluidic approaches offer deterministic control over the cell encapsulation process, they require complex instrumentation involving hydrodynamic focusing and flow control for tracking multiple cells in these systems [26], [27], [28], [29]. The drop-on-demand based approaches that achieve high cell viability use larger volume droplets than the cell size. This makes the encapsulation process random, since cell encapsulating droplets are generated from a reservoir containing a cell suspension. On the other hand, they offer simultaneous encapsulation, isolation and patterning of cells in a single process step, which is desirable for handling sensitive cell types and for applications that demand patterned cells after sorting. Further, these statistical encapsulation systems do not become more complicated, as the number of cell types increase in the heterogeneous solution. Several statistical approaches were presented for encapsulation using various technologies including cell encapsulation in polymers [16] and emulsions [17], cell separation with micro-well arrays [30], fluorescence-activated droplet sorting [26], and droplet generation using microfluidics [27], [28], [29]. Villani et al. presented a statistical approach in alginate membrane formulation for cell encapsulation [16]. Using a passive tool, e.g., a microwell template, Love et al. experimentally analyzed manual cell loading efficiency for microwells with homogeneous cell types [30]. Abate et al. also presented a close-packed droplet generation in a closed channel and flow-focus to enhance random encapsulation efficiency with control over the flow and feedback encapsulation signal in a microfluidic channel [27].

Recently, microchip technologies have created multiple new avenues through experimental studies to isolate, capture, pattern cells in microscale fluidic volumes impacting a variety of fields. However, the emphasis has been on engineering, device modeling and medical applications, whereas the statistical analysis of such events has fallen short of focus. Among these microfluidic manipulation technologies, cell encapsulation processes within microscale droplet volumes have not been theoretically investigated from a statistical or stochastic point of view for droplet ejectors. In this paper, we statistically modeled and experimentally analyzed random cell encapsulation processes in microdroplets.

## Methods

To model the encapsulation process, we assumed that droplets were generated from a heterogeneous cell suspension consisting of target and non-target cells. The cell encapsulation process can be described by using four random variables with respective probability distribution functions (PDFs). These random variables are: number of droplets containing cells, number of cells per droplet, number of target cells per droplet, and number of droplets containing only a single target cell. Employing different loading cell concentrations and target cell concentrations, each random process can be characterized by the rate of empty droplets, average number of cells per droplet, number of target cells in the printed droplets, and the rate of single target cell encapsulation, respectively. The PDF corresponding to each random variable can be used to estimate process characteristics based on experimental results. We followed four steps to analyze our single target cell encapsulation process: (i) we defined our system as a set of stochastic processes with random variables, (ii) estimated the minimum number of droplets at which the variables follow (approximately) a normal distribution, and determined whether the suggested processes are biased or un-biased, (iii) established statistical models and parameters for each random process, e.g., mean (μ), variance (σ), and Poisson distribution parameter (λ) for the cell encapsulation process, and (iv) evaluated overall system efficiency to find and encapsulate single target cells using central limit theorem (CLT) under conditions of simple random sampling (SRS).

### 1. Definition of cell encapsulation process with random variables

**Figure 1:**
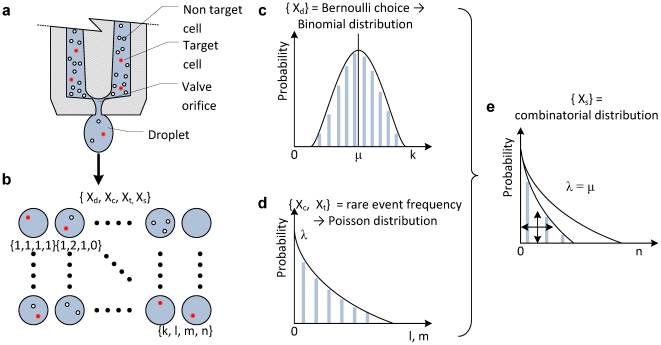
Schematic notation of random variables and probability distribution functions for statistical analysis. A heterogeneous solution of target and non-target cells is loaded to a droplet ejector. (**a**) Random cell encapsulation process. (**b**) Three random variables and one dependent variable were mapped to a patterned array of cell encapsulating droplets which represents number of droplets that contain cells, number of cells per droplet, number of target cells, and droplets encapsulating a single target cell, respectively. The probability of the process can be described as (**c**) binomial distribution, which represents success and failure corresponding to cell containing and empty droplets, respectively. (**d**) Poisson distribution is used for the random variable, X_c_, since the number of cells per droplet is the count of occurrence of a rare event (i.e., probability of the event is very low) in probability space with respect to the number of sampled droplets and droplet volume. (**e**) Overall system random process becomes the combined function of suggested PDFs. The PDFs for the random variables, X_d_ and X_c_, are used for the overall PDF of the system. The parameter λ represents the Poisson coefficient, and μ, and σ represent mean, and variance of the underlying probability distributions, respectively. All distribution functions were interpolated to a continuous curve with colored bars on graph indicating the discrete values.

Random variables for cell encapsulation process in droplets were schematically shown in **Fig. 1a, b** and the setup was described in detail in **Supporting Information S1A** and **Fig. S1**. A heterogeneous solution of target and non-target cells is loaded to a droplet ejector. Three random variables represent number of droplets that contain cells, number of cells in a droplet, and number of droplets encapsulating only a single target cell, which are denoted as X_d_, X_c_, and X_t_, respectively, and are defined at the sampling space from droplet array (n = 100 droplets forming a 10×10 array of patterned droplets). These random variables are proposed to have three probability distributions, Binomial, Poisson and a combined distribution, respectively as shown in **Fig. 1c–e**.

The cell encapsulation process in droplets can be classified into two discrete random processes: (i) random cell encapsulation in droplets from a homogeneous cell mixture, and (ii) encapsulation of a single target cell from a heterogeneous cell mixture. The single target cell encapsulation could be modeled resulting from an ensemble effect combined with a high probability of cell encapsulation using Binomial distribution, and a rare probability event process that follows Poisson distribution as shown in **Fig. 1c and 1d**. A binomial process yields the random variable, X_d_, which gives the number of successes in *n* Bernoulli trials. In a Bernoulli trial, “success” is the occurrence and “failure” is the non-occurrence of the desired event. In our setup, “success” is the event of a droplet containing cell(s), and “failure” is the event of a droplet being empty. The random variable, X_c_, is expected to follow a Poisson distribution, since the number of cells per droplet is a rare event in discrete probability space. The overall system PDF can be obtained by combining PDFs of the two independent random variables, X_d_ and X_c_ and each PDF is shown in **Fig. 1e**. The number of droplets containing a single target cell, X_s_, is a variable that depends on two other variables, namely, number of droplets containing cells, and number of target cells in a droplet. The parameters for each PDF can be estimated by using experimental results for independent random variables in discrete probability space, i.e., in the space of ejected cell encapsulating droplets.

### 2. Determination of the sample size for normal approximation

We have determined the required sample size that would render the normal approximation appropriate for our analysis. In this study, cell encapsulation was validated as a random process provided that it satisfies experimentally the conditions for the law of large numbers (LLN) for the normal approximation assumption to hold. As more details are provided in **Section 4**, the sampling number to fulfill LLN for the modeled process can be estimated by predetermined confidence and tolerance levels (**Table S3 & S4**). In our experiments, we picked 90% for confidence level and 15% for tolerance level, which are commonly used values in the literature [31], [32]. The theoretical condition for LLN follows the below inequality [33], [34]:
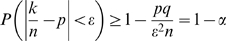
(2.1)where P() stands for the probability, k for number of successes, n for sampling number (i.e., sample size), ε for tolerance, 1−α for confidence level and p for probability of success in a single trial or event. The inequality indicates the required sample size, n, for a relative frequency to deviate from the probability of an event at a certain confidence and tolerance level. Therefore, the minimum required sample size, n, would be:
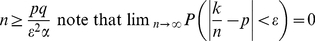
(2.2)A stronger result is provided by the strong law of large numbers, which states the convergence of k/n to p with probability one in case of sufficient number of sampling [33], [34].

### 3. Random process modeling

The cell encapsulation process follows a sequential process based on Bernoulli trials to find a successful case. Binomial process represents how many times the sequential process will be a success (i.e., how many of the droplets contain cell(s)) regardless of the number of cells per droplet. Similarly, when the successful case is rare, the probability of encapsulating homogeneous target cells shows Poisson distribution as a special case of Bernoulli trials [33], [34]. Therefore, the overall process of finding droplets that contain a single target cell among droplets that contain patterned heterogeneous cells becomes a combination of the two processes. Here, we verify that our proposed platform follows a binomial distribution for cell encapsulation, Poisson distribution for homogeneous cell encapsulation, and a combined probability distribution for single target cell encapsulation process based on experimental results.

PDF of binomial distribution is discussed in **Supporting Information S1B**. To compute the probability distribution requires that a certain event occurs k times in a sequence of n events. In a discrete probability space, this PDF can be described with the following formula (eq. 3.1). For the random variable, X_d_, number of droplets that contain cells, is associated with the occurrence of k successes in n trials [34]:
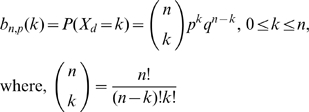
(3.1)The mean and variance of the binomial distribution, b_n,p_(k), are μ = np and σ^2^ = npq. From the computational perspective, the fast growth of the factorial function is often handled by the Stirling approximation formula in the binomial probabilities [35]:
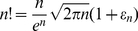
(3.2)The quantity, tolerance (ε_n_), tends to zero as n goes to infinity. The convergence is rapid (e.g., n≥20), where the error of the approximation is below 0.5%.

Since high cell concentrations might have lower probability for single cell encapsulation compared to low cell concentrations, the low cell concentration and cell volume fraction can be represented with a Poisson distribution for encapsulation of “*a single cell*” in a droplet. The probability distribution can be computed with the following equation in discrete space (the derivation of Poisson distribution is discussed in **Supporting Information S1C**) [34]:
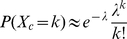
(3.4)where n is number of droplets, P is probability for the number of cells in a droplet, and k is the number of cells in a droplet. The μ, σ, and λ values for Poisson distribution have the following relations with the average and variance [34]:
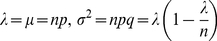
(3.5)where n is the number of all cells per droplet.

In the case of encapsulating a single target cell in a droplet from a heterogeneous cell mixture, the probability to encapsulate a single target cell becomes lower than the probability for a homogenous cell mixture. So we attempt both a binomial distribution and a Poisson distribution model for different cell loading concentrations. Finally, overall process PDF for a single target cell encapsulation was modeled using two PDFs: the cell encapsulation process (modeled as binomial distribution) and single target cell encapsulation process (modeled as Poisson distribution). These PDFs were combined to make a single probability function describing the complete process. The PDF of the random variable X_s_ can be obtained by combining the PDFs of the two random variables, X_d_ and X_t_, (see **Table S1**). Notice that X_t_ cannot be positive when X_d_ is not positive, in the sense that X_d_ = 0 forces X_t_ = 0. However, conditional on X_d_>0, it is reasonable to assume that X_d_ and X_t_ are independent, and hence PDF of X_s_ can be written as follows:
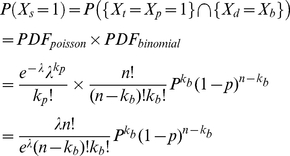
(3.6)where k_b_ and k_p_ stand for the number of occurrences for the binomial and Poisson distribution, respectively. Note also that P(X_s_ = 0) = 1−P(X_s_ = 1).

### 4. System evaluation using CLT and simple random sampling (SRS)

Following CLT (as described in **Supporting Information S1D**), single cell encapsulation in a droplet as random variable X and any positive real constant k follows the Chebyshev's inequality [33]:
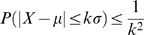
(4.1)Based on this inequality, we can estimate the conditions with a certain confidence level following CLT based on a simple random sampling process. The confidence level can be determined following the conditions of the experiment or user preference according to the type of application. For cell encapsulation model, we chose 90% confidence level, (hence 1−α = 0.90, which implies α = 0.10), and 15% tolerance, ε, for lowest probability (p = 0.5) for the number of sampled droplets. LLN was shown in **Table S3** using a patterned array of 10×10 droplets (i.e., n = 100 droplets) for different cell loading concentrations. First, randomness of process was verified by three variables, number of droplets (n), tolerance (ε), and confidence level (1−α) using eq. 4.1. Following the LLN, minimum sample size was estimated to be 100 droplets for 90.0% confidence level with 15.0% tolerance. This sampling volume of a droplet represented 0.76% of the total volume of the ejection reservoir (0.1 ml). Random processes have different PDFs in accordance with their parameters, i.e., number of droplets that contain cells (*X_d_*), number of cells in a droplet (*X_c_*), number of target cells per droplet (*X_t_*), and the number of droplets that contain single target cells (*X_s_*). These random variables are represented by two PDFs to statistically model the cell encapsulation process, i.e., binomial and Poisson distributions. We investigated probability values and parameters, λ, for each case with respect to the cell loading concentrations, cell volume fraction, and percentage of target cells. In the case of simple random sampling (SRS) process, statistical characteristics of a relatively small sampling volume could represent the characteristics of a large population based on CLT. In our experiments, the target cell fraction (*F*_%_) shows same concentration as the reservoir concentration for 10.0% to 50.0% at 1.0×10^5^ cells/ml concentration (C_opt_) under conditions of 90.0% confidence level and 15.0% tolerance.

## Results

**Figure 2:**
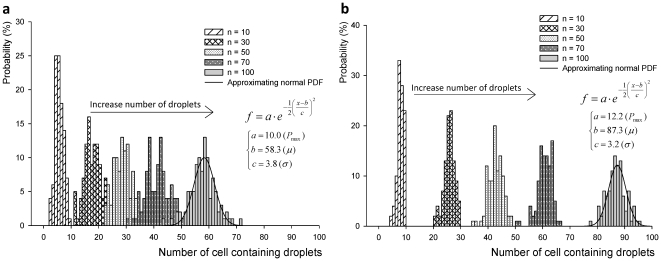
LLN (Law of large numbers) for different sampling numbers from *n* = 10 to 100 was shown according to cell loading concentrations (a) 1.0×10^5^ cells/ml and (b) 2.0×10^5^ cells/ml. Probabilities of cell encapsulation in droplets are P(X_d_ = 1) = 58.3% and 87.3%, respectively. As number of droplets increase, high cell concentration, 2×10^5^ cells/ml, also follows normal distribution.

For cell encapsulation model, 90% confidence level, (1−α), and 15% tolerance, ε, for lowest probability (p = 0.6) were used to determine the number of ejected droplets encapsulating cells. LLN was shown by investigating the PDF shape with 1.0×10^5^ cells/ml to 2.0×10^5^ cells/ml cell loading concentrations. As the sampling number of random variable, X_d_, increases from n = 10 to 100, the shape of its PDF, which actually is a binomial PDF, gets closer to a normal distribution as indicated in **Fig. 2**. Experimental results showed that the probability of cell encapsulation in droplets was P(X_d_ = 1) = 58.3% and 87.3% for the cell loading concentrations of 1.0×10^5^ cells/ml to 2.0×10^5^ cells/ml, respectively. The PDF curve shows that the results follow normal PDF which has 58.3±3.8% for average and standard deviation with a 10% maximum probability of cell encapsulation at 1.0×10^5^ cells/ml concentration (**Fig. 2a**). As sampling number increases, high cell concentration, 2.0×10^5^ cells/ml, also follows normal distribution (**Fig. 2b**). Average probabilities for different cell loading concentrations range from p = 0.87 to 0.58, which require 50 to 108 sampled droplets. Following these results, the sampling number, n = 100 droplets (selected as a 10×10 droplet array from the entire set of printed droplets), seems to be sufficient for the droplet based cell encapsulation process to follow approximately a normal distribution. The array size was also suitable based on the field of view of the detection equipment such as a microscope or a lensless imaging system, (i.e., 10×10 array of droplets for the allowable field of view for imaging with CCD systems, 30 mm×40 mm size). Using 100 instead of 107 suffices for an approximation to normal distribution as presented in **Figure 2**.

**Figure 3:**
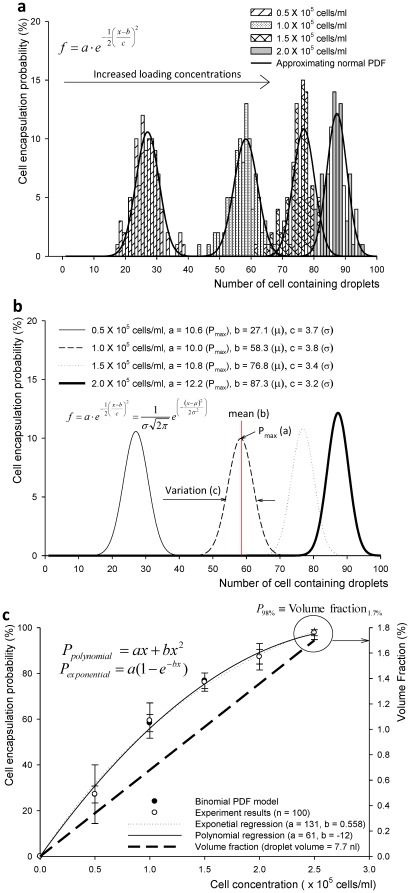
Probability distribution functions of Bernoulli's random variable, X_d_. (**a**) Binomial distribution functions (n Bernoulli trials for discrete random space) are shown with fitted PDF curves. The mean values of modeled binomial distribution (**b**) 27.1%, 58.3%, 76.8%, and 87.3% for probability of cell encapsulation at cell loading concentrations of 0.5×10^5^, 1.0×10^5^, 1.5×10^5^, and 2.0×10^5^ cells/ml, respectively (*n*_test_ = 100 droplets). Exponential regression curves fit the experimental results (coefficients of exponential regression: a = 131, b = 0.558, R^2^ = 0.995). (**c**) Cell encapsulation probability and volume fraction (which is the ratio of cell volume divided by droplet volume, 7.7 nl) are shown as a function of cell concentrations. At cell concentration 2.5×10^5^ cells/ml, probability of cell encapsulation was 98.0% and the volume fraction was 1.7% (which represent the cell loading concentration and the minimum droplet volume to encapsulate a single target cell with the proposed mechanical valve system, respectively). In summary, 1.7% cell volume fraction is the optimal value to achieve a very high cell encapsulation probability.

The formula in (eq. 3.2) was utilized to calculate the fitted PDF curves using MATLAB® (Version R2010a, The MathWorks, Inc, MA). **Fig. 3a, b** shows the experimental results and approximating normal curves for the PDFs of Binomial process. In fact, we plot the discrete PDF functions together with an approximating continuous curve (treating the discrete variables as if they were continuous) for better visualization throughout the article. To fulfill the condition for a large sampling set, we tested n = 100 droplets as described earlier in **Section 2**. Our experimental results followed the binomial distribution when the number of droplets that contain cells ranged from 1.0×10^5^ cells/ml to 2.0×10^5^ cells/ml as shown in **Fig. 3a**. Following the binomial distribution model, for the cell loading concentrations 0.5×10^5^, 1.0×10^5^, 1.5×10^5^, and 2.0×10^5^ cells/ml, the probabilities of cell encapsulation were 27.1%, 58.3%, 76.8%, and 87.3%, respectively. These probability values are estimated from the means of the empirical PDFs shown in **Fig. 3b**.

In the random process, the physical cell encapsulation process is affected by the ejection mechanism and cell loading concentrations. In **Fig. 3c**, the relationship between cell encapsulation probability and volume fraction was shown as a function of cell concentration. The cell encapsulation probabilities showed exponential increase as cell concentration increases (**Table S4**). We observed that the binomial distribution fits our experimental results (**Fig. 3** **)**. At a cell concentration of 2.5×10^5^ cells/ml, probability of cell encapsulation was as high as 98.0%. This result represented that small cell volume to droplet volume ratio was needed to encapsulate cells within a large media droplet volume. For 100% cell encapsulation probability, minimum droplet volume and required cell concentration was calculated as 30.8 picoliter (pl) and 32.5×10^6^ cells/ml by the following equation:
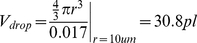
(3.3)Therefore, at volume ratio value of 1.7% (i.e., 0.017) the droplet generation system encapsulates cells with a minimum droplet volume of 30.8 pl. This volume fraction corresponds to maximum cell loading concentration of 32.5×10^6^ cells/ml with a droplet volume of 7.7 nanoliter (nl). Exponential regression curves fit the experimental results (the adjusted R^2^ value is 0.995). The coefficients of exponential regression are a = 131 and b = 0.558 as shown in **Fig. 3c**. These results indicate that cells get encapsulated in droplets with a higher probability at 2.0×10^5^ cells/ml than 1.0×10^5^ cells/ml. However, the cell concentration of 1.0×10^5^ cells/ml showed higher probability of “*a single cell per droplet*” than that of 2.0×10^5^ cells/ml. Since only volume fraction of 1.7% is required for encapsulating a cell with over 98% probability, high cell concentrations might have lower probability for single cell encapsulation compared to low cell concentrations. The low cell concentration and cell volume fraction can be represented with a Poisson distribution for encapsulation of “*a single cell*” in a droplet as in (eq. 3.4).

**Figure 4:**
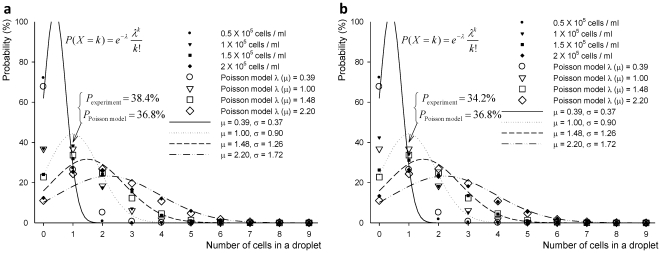
Probability distribution functions for the number of cells per droplet, X_c_. In the case of droplets containing a single cell, the Poisson distribution agrees with the experimental results since the probability becomes small. Experimental results and modeled values as Poisson distribution for single cell encapsulation process agrees with ±2% error at 1.0×10^5^ cells/ml (*np* has a moderate size, λ = 1.0 for *n* = 10 cells in a droplet) for (**a**) 10% and (**b**) 50% target cell mixture, *n*_test_ = 100 droplets. The maximum probability and PDF is not affected by cell loading concentrations. The curves are generated using the Poisson distribution instead of the binomial distribution in continuous random variable space (λ = μ = *np*, σ = *npq* = λ(1−λ/*n*)). In spite of the fact that 1.5 and 2.0×10^5^ cells/ml cell concentration show higher cell encapsulation probability as shown in Figure 3, highest probability for single cell encapsulation is achieved at the specific cell concentration of 1.0×10^5^ cells/ml corresponding to 1.0% of volume fraction. The result is obtained from the peak points of each PDF, which gives highest probability of X_c_ = 1.

**Fig. 4** shows the PDF of the number of cells in a droplet, P(X_c_ = k). In the case of droplets containing a single cell, the Poisson distribution matches the experimental results as the occurrence of “single cell encapsulation” becomes rare. That is, the number of droplets containing cells can be modeled as a binomial process, and the same holds for the number of droplets that have single target cells. However, the latter seems to be closer to a Poisson distribution, since the probability of success (i.e., probability of a droplet having a single target cell) is low and the number of droplets is large. Moreover, the results showed the same probability distribution regardless of target cell concentrations for 10% (**Fig. 4a**) and 50% (**Fig. 4b**) target cell mixture with *n*_test_ = 100 droplets (0.76 µl total sub-sampling volume, single droplet volume is 7.6 nl). Experimental results and modeled values of Poisson distribution parameters match with ±2.0% error from 10.0% to 50.0% cell loading concentration at 1.0×10^5^ cells/ml.

In **Fig. 4**, the PDF curve for each experiment is calculated by using (eq. 3.5) using the Poisson approximation to binomial distribution. This is also plotted as a continuous random variable for convenience in visualization. At lower cell concentrations, say 0.5×10^5^ cells/ml, we observed Poisson distribution, since the probability for encapsulating cells in a droplet is very low. The other distributions for the concentrations are closer to the binomial distribution than Poisson distribution due to higher cell concentration and higher cell encapsulation probability. Following the experimental results and the statistical model as shown in **Fig. 4**, P_experiment_ and P_Poisson model_, 1.0×10^5^ cells/ml was indicated as an optimal concentration to encapsulate a single cell in a droplet for our cell printing platform, since it has the highest single cell encapsulation probability. Even though, 1.5×10^5^ and 2.0×10^5^ cells/ml cell concentration show higher cell encapsulation probability as shown in **Fig. 3**, highest probability for single cell encapsulation at 1.0×10^5^ cells/ml corresponding to 1.0% of volume fraction. The highest probability estimates are obtained from the peak points in each PDF, which gives highest probability for X_c_ = 1.

**Figure 5:**
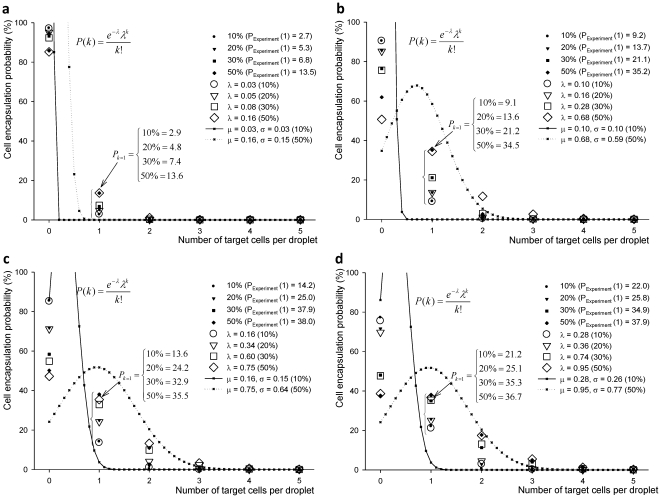
Probability distribution functions for encapsulation of target cells from (a–d) 0.5×10^5^ cells/ml to 2.0×10^5^ cells/ml cell concentrations, P(X_t_). The PDFs are based on experimental results, Poisson distribution, and binomial distribution treating the variables as continuous (e.g., it is not possible to have 0.1 cells, but we estimate the cell encapsulation probability for this value), for cell loading concentration (**a**) 0.5×10^5^ cells/ml, (**b**) 1.0×10^5^ cells/ml, (**c**) 1.5×10^5^ cells/ml, (**d**) 2.0×10^5^ cells/ml.

As for the case of encapsulating a single target cell in a droplet from a heterogeneous cell mixture, **Fig. 5a–d** shows PDFs, P(X_t_), for heterogeneous cell mixture with different cell loading concentrations from 0.5×10^5^ cells/ml to 2.0×10^5^ cells/ml. The shape of Poisson distributions were determined based on experimental results. Binomial distributions and Poisson distribution for different cell loading concentrations were plotted in both the discrete space and continuous random variable space. As shown in **Fig. 5**, each PDF resembles a Poisson distribution as the percentage of target cells and homogeneity decreases in cell mixtures.

**Figure 6:**
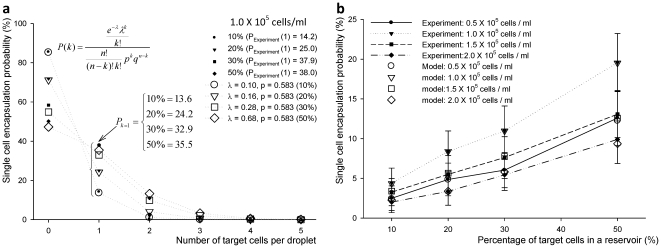
The plot of single cell encapsulation probability versus number of target cells per droplet (a), and percentage of target cells in a reservoir (b) PDFs for a single target cell encapsulation, P(X_s_) were shown with combined PDFs for selected cases: (a) Poisson distribution for 1.0×10^5^ cells/ml cell concentration for four different target cell concentrations, and (b) cell encapsulation probability compared with experimental results from 10% to 50% target cell mixture. Modeled PDFs showed 5% error compared to the experimental results using specific parameters, μ, σ, and *p*.

For the overall process PDF for a single target cell encapsulation as in (eq. 3.6), **Fig. 6** shows PDFs for the random variable, X_s_, and conditional PDF for selected cases of 1.0×10^5^ cells/ml (**Fig. 6a**) and 10% target cell mixture (**Fig. 6b**). The combined PDF is similar to Poisson distribution, since the overall probability of single target cell encapsulation (considering empty droplets) is also low. The error in the fit of the modeled Poisson PDFs is within 5%, when the experimental results are compared using specific parameter values of μ, σ, and p.

Based on the suggested model on (eq. 3.6), the number of homogeneous droplets was modeled using the Poisson distribution. The single target cell encapsulation process in a droplet can be modeled following two control parameters: cell loading concentration and percent mixture of target cell. Average number of cells, λ, for four different cell loading concentrations and target cell concentrations were determined as shown in **Table S2**. As cell loading density increases, target cell concentration, λ values increase, λ_max_ = 0.95 and λ_min_ = 0.03. Based on these experimental and analysis results, statistical models can be determined based on λ values, e.g., λ = 0.05 for 1.0×10^5^ cells/ml with 10% target cell mixture.

## Discussion

For certain applications, for instance in tissue engineering and/or high throughput testing [17], [30], [36], patterning cells is also an important step that needs to follow the cell sorting step in microfluidic systems. These systems will still require an additional step for sorted cells to be separated to access individual target cells and for patterning. The droplet based encapsulation approach offers the advantage to sort and spatially pattern target cells at specific predetermined locations simultaneously compared to other approaches such as microfluidics, which sort and place cells separately or use presorted cells. The droplet based system can be advantageous, since it has fewer handling steps, which can be important when dealing with various cell types. Hence, the methodology that we are offering is unique in the sense that it can work with both sorted and non-sorted cells depending on the application. Further, drop-on-demand encapsulation process is suitable for cell sorting and encapsulation simultaneously. In case of a heterogeneous sample, microfluidic approach may require that each target cell should be tracked during cell sorting process and be patterned through an additional process step. Along with the cell sorting process, we introduced statistical modeling and patterned droplet arrays which are placed onto a surface with spatial control using a drop-on-demand cell encapsulation system. When multiple kinds of cells are present in a heterogeneous sample, the statistical approach would efficiently select target cells. The encapsulation approach does not require individual tracking of cells as compared to the microfluidic approaches, where cells flow in channels for sorting. The direct encapsulation from the heterogeneous solution could offer to keep the system complexity low.

When performing cell encapsulation studies, commonly observed experimental conditions in cell culture needs to be taken into account, such as the aggregation and settling of cells in the suspension form. In our studies, cell encapsulating droplet generation took place within minutes after the cell suspension was prepared. Therefore, cell settling or aggregation of the cells in the reservoir was not considered to have a significant effect on the results. Various mixing methods, such as magnetic stirring, can be used to prevent cell aggregation and settling for experiments lasting longer durations. On the other hand, in our earlier work, we have shown that different fluid types with different rheological properties can be used to generate droplets and encapsulate cells [2], [8], [37], [38]. These studies have indicated that the system presented here can produce uniform sized droplets with high repeatability for a wide range of viscosity values with different cell concentrations, including non-Newtonian fluids.

Encapsulation of a few to many cells in micro-scale droplets has been investigated for applications in tissue engineering, in which cell-encapsulating hydrogels can be used as building blocks for generating organized tissue structures. In these studies, the control over the number of cells in hydrogel building blocks (e.g., microscale droplets) is essential, where the number of cells per building block determines the overall cell density in the resulting tissues, and hence the structural and functional outcome [39]. Therefore, for applications in tissue engineering, it is critical to achieve controllable cell density in small volume hydrogel droplets for achieving reproducible and effective results.

Cell clusters (e.g., pancreatic islets) are currently an important research area, which has the potential to offer alternative treatments for diseases such as, Type-1 diabetes [40]. Cell clusters can be considered as small organs, which are composed of multiple cell types in a complex three dimensional organization. Encapsulation of multiple cells, multiple cell types and cell clusters in droplets with a deterministic approach would present unique challenges, which warrant further investigation. It should be noted that many cell types depend on supporting populations of other cells and cell types to survive. Therefore, a detailed investigation on encapsulation of multiple cell types that can serve as side populations is needed. The present methods and models in this manuscript can be modified to account for encapsulation of multiple cell types, which would support applications where supporting cell populations are important.

The presented drop-on-demand approach for single cell sorting has a trade-off from a deterministic cell encapsulation aspect compared to the microfluidic cell encapsulation approaches. For single cell encapsulation, microfluidic method provides a more deterministic method and better control over cell encapsulation. However, as the heterogeneity of sample increases, the types of cells that need to be tracked in the sample also increases leading to incline in complexity of microfluidic systems due to more complex peripheral setups and high-end computerized controls [41], [42]. The deterministic microfluidic approach offers an efficient system to handle single to few cells. Further, microfluidic approach can be convenient in case an integrated on-chip experiment requires further cell handling steps post-sorting such as on-chip polymerase chain reactions (PCR). Finally, throughput of drop-on-demand sorting depends on the parallel printing setup and the wide field of view imaging. On the other hand, the drop-on-demand cell encapsulation approach here offers the benefit of fewer handling steps to sort and pattern cells, where cells might be affected by the conditions in the physical environment (e.g., stem cells and oocytes). Further, by characterizing the performance of the system statistically, the results can be repeatable and controlled such that the system would be used as a biotechnological tool to separate cells for specific applications, where minimal cell handing could be an important issue. For instance, the importance of cell microenvironment on cellular function has been demonstrated [12], [13], [43], [44], [45], [46]. This also points to the direction to minimize handling steps and handling time for live cells. Another challenge in microfluidic cell encapsulation is direct accessibility to cells. In microfluidic systems, cells are located in closed channels, which might make them harder to access directly. There is a need for an additional droplet placement step, especially when spatial patterning is needed to directly access cells. By the encapsulation approach that we present here, target cells can directly be accessed in patterned droplets without going through additional processes.

We investigated the cell encapsulation process in microdroplets. We modeled the encapsulation process of a mechanical valve system that randomly encapsulates target cells from a heterogeneous cell suspension. Using four random variables and corresponding probability distribution functions (PDFs), the cell encapsulation process was described and used to estimate process characteristics such as mean (μ), variance (σ), and Poisson distribution parameter (λ) for different cell concentrations and target cell mixtures. These models exhibited Poisson distributions with 16 different values of a parameter as shown in **Fig. 5** and **Fig. 6**. The single target cell encapsulation process also followed Poisson distribution and matched with 90.0% confidence level and 15.0% tolerance to experimental results. These results show droplet ejector system can be used to encapsulate single target cells at concentrations as low as 8.0% to 9.0% by using a patterned array of 10×10 droplets. If the size of droplet array follows law of large numbers (LLN) as a sampling set, then the statistical information of entire sample set for cell encapsulation can be determined based on central limit theorem (CLT). These results were demonstrated under the conditions of simple random sampling (SRS).

In conclusion, statistical and stochastic modeling proves to be a powerful and promising tool to determine the conditions for single target cell encapsulation. In this article, we have theoretically analyzed the encapsulation of a single target cell in microdroplets from heterogeneous cell mixtures and supported our theoretical results with experimental data. This analysis explains the statistical dependence of encapsulation of single cells in droplets.

## Supporting Information

Supporting Information S1 (DOC)

Figure S1 **Image and illustration of a drop-on-demand cell encapsulation system.** (**a**) Image of the setup. A computerized xyz stage was synchronized with a pulse controller, Labview™. The automated stage positioned the substrate with 5 µm spatial resolution. A 10× magnifying camera permitted in-situ imaging of the droplets. (**b**) Schematic of droplet ejector showed cells flowing into the valve driven by a controlled air pressure pulse. A heterogeneous sample, mixture of cells and media solution, was loaded into a reservoir. Each droplet was placed at a predetermined position (10×10 droplet array).
(TIF)

Table S1 **Abbreviations and descriptions for statistical modeling of single target cell encapsulation.**
(DOC)

Table S2 **Random variables in our data set and representative meanings of each variable for random cell encapsulation process.** Three different random variables (i.e., *X*_d_, *X*_c_, and *X*_t_) were defined in discrete independent domain and one dependent variable (i.e., *X*_s_) was defined by a combination of independent variables for overall process efficiency. Three variables were used to represent percentage of empty droplets, effect of number of cells in droplets as a function of loading cell concentrations, and target cell concentrations, respectively.
(DOC)

Table S3 **Values of Poisson coefficient, λ, for four different percentage of target cell mixture and four different cell loading concentrations at reservoir.** Probability of encapsulating a single target cell in a droplet was presented by probability distribution function, P(*X*_s_). The number of homogeneous droplets was modeled using Poisson distribution in a random variable space, i.e., number of target cells. The model was verified using a coefficient, λ, and experimental results. Average number of cells, λ, for four different cell loading concentrations and target cell concentrations were determined. As cell loading density increases, target cell concentration, λ values increase, from λ_min_ = 0.03 to λ_max_ = 0.95. Based on these experimental and analysis results, statistical models can be determined based on λ values, e.g., λ = 0.10- for 1.0×10^5^ cells/ml with 10% target cell mixture.
(DOC)

Table S4 **Statistical modeling results for drop-on-demand single target cell encapsulation.** (**a**) Randomness of process was verified by three variables, number of samples (n), tolerance (ε), and confidence level (1−α) using an inequality^(**)^. Following the law of large numbers (LLN), minimum sample number was determined as 100 droplets for 90.0% confidence level and 15.0% tolerance. This sampling volume of a droplet represented (0.76 µl = 10×10×7.6 nl) 0.76% of the total volume of the ejection reservoir (0.1 mL). (**b**) Random processes have different PDFs in accordance with their parameters, i.e., droplets that contain cells (*X_d_*), number of cells in a droplet (*X_c_*), number of target cells per droplet (*X_t_*), and droplets that contain single target cells (*X_s_*). These four random variables are represented by binomial, Poisson, and normal distributions to statistically model the cell encapsulation process. We investigated probability values and parameters, λ, for each case with respect to the cell loading concentration, cell volume fraction, and percentage of target cells. (**c**) In the case of SRS process, statistical characteristics of a random sampling volume can represent the characteristics of a large population, and the required number of droplets (i.e., sample size) is based on the CLT. In our experiments, the target cell fraction (*F*%) shows same concentration as the reservoir concentration for 10.0% to 50.0% at 1.0×10^5^ cells/ml concentration (C_opt_) under conditions of 90.0% confidence level and 15.0% tolerance.
(DOC)
