# Supplementary material for: Statistical Modeling of Single Target Cell Encapsulation
Source: PLoS One. 2011 Jul 21;6(7):e21580. doi: 10.1371/journal.pone.0021580 (PMC3140975; doi:10.1371/journal.pone.0021580)
Supplement: Supporting Information S1 — (DOC) [file pone.0021580.s001.doc]

**Supporting Information S1**

Statistical modeling of single target cell encapsulation

SangJun Moon^a^, Elvan Ceyhan^b^, Umut Atakan Gurkan^a^ , and Utkan Demirci^a,c*^

^a^ Demirci Bio-Acoustic-MEMS in Medicine (BAMM) Laboratory, Center for Bioengineering, Brigham and Women's Hospital, Harvard Medical School, Boston, MA, USA

^b^ Department of Mathematics, Koc University, Istanbul, Turkey

^c^ Harvard-MIT Division of Health Sciences and Technology, Massachusetts Institute of Technology, Cambridge, MA, USA

*Corresponding author: [udemirci@rics.bwh.harvard.edu](mailto:udemirci@rics.bwh.harvard.edu)

***Drop-on-demand cell encapsulation setup:*** Drop-on-demand cell encapsulation system can generate droplets both with spatial control over position and temporal control over ejection. The single cell droplet ejector system consists of an automated xyz stage (NLS4 Series Precision Linear Stage, Newmark systems Inc.) and a sub-nanoliter dispensing valve (TechElan LLC, G100), which were synchronized with a control program, Labview^TM^ (Labview, National Instruments Corporation) as shown in **Figure S1**. The spatial resolution and repeatability of the stage are 0.13 µm and 5 µm, respectively. To image *in-situ* cell encapsulating droplets during the ejection process, a CCD camera (Edmund optics, EO-1312M) equipped with a 0.5× lens (INFINITUBE FM-200 (NT58-309, 2×) and a PL/FD-195 OBJ objective (NT59-115, 0.25×, Edmund optics) was combined with a synchronized light controller (S4000 strobe controller, Edmund optics) for stroboscopic illumination. The overall system, i.e., xyz stage, sub-nanoliter dispensing valve, and stroboscopic light controller, was synchronized and programmed by a PC-based DAQ control board (NI cDAQ-9172 and NI-9401**,** National Instruments). Pressure regulated nitrogen gas was connected to a syringe reservoir through an adapter cap (KDS503S6, Techni-Tool Inc.), and the syringe was connected to the sub nanoliter dispensing valve by tubing (Tygon® tube, Fisher scientific Inc.).

The mechanism of ejection is based on a droplet generator that uses a square electrical pulse to control a solenoid valve. As the valve opens the cell encapsulating droplets are pushed through the ejection opening by the air pushing on the reservoir. When the valve closes the droplet generation is completed and cells encapsulated in droplets are ejected. The width of the electrical pulse determines how long the valve will stay open and how big the droplet size will be as these operational parameters of the technology are earlier described [1].

**A. Definition of cell encapsulation process with random variables**: It is important to understand the mechanism of the cell encapsulating process to be able to control the number of cells per droplet. Statistics is widely adapted to biological analysis since number of cells is typically large enough to follow the law of large numbers (LLN) and sampling processes are random, i.e., they abide the simple random sampling (SRS) process [2]. Following LLN, a large sampling set satisfying SRS process shows that probability distribution of a random variable approximately follows normal distribution [3]. Under these conditions, the statistical characteristics of an entire population can be estimated by only using a small sample based on SRS together with central limit theorem (CLT) [4].

The random processes of a system can be defined with random variables and probability density functions (PDFs) as functions of each random variable. A random variable is a function that takes an input from a domain set to an element in a target set (randomly). In a random experiment, some of the PDFs can be a combination of other PDFs in the probability space (*E*, *A*, *P*), where *E*, *A*, and *P* represent set of all events (i.e.*,* universe), set of events, and probability of an event, respectively. So a PDF here can be a random variable with domain being the set of all possible random processes, and target being the set of PDFs that might fit these processes. For the cell encapsulation process, three random variables were defined at each independent probability space, i.e.*,* number of droplets that contain cells, number of cells in a droplet, and number of droplets that contain a single target cell as shown in **Tables S1 and S2**. The number of droplets that contain cells, X_d_, is defined to evaluate empty droplet rates during cell encapsulation process and is expected to follow a binomial distribution in a discrete probability space. The number of cells in a droplet, X_c_, depends on the cell concentration levels at the reservoir and is expected to follow Poisson distribution. In the case of a heterogeneous cell mixture, the number of target cells per droplet, X_t_, showed the degree of droplet array homogeneity when compared to X_c_ and indicates the relation of target cell concentration between the droplet array and in the reservoir. Finally, the number of droplets that contain a single target cell, X_s_, is a variable that depends on two other variables, namely, number of droplets that contain, and number of target cells in a droplet. Furthermore, X_s_ represents an overall statistical efficiency of single target cell encapsulation process, which is the goal in our experiments.

These random variables were defined at three probability spaces with different PDFs. In continuous probability space, the PDF of a random variable $X$ is defined as $F_{X}(x)\leq P\left(X\leq x\right)$ with probability values for each case. For discrete random variables, with probabilities $P_{i}$ assigned to the singleton events of$A_{i}=\{x=x_{i}\},$the following holds:

|  | $F_{X}\left(x_{i}\right)\equiv F(x_{i}) \leq\sum_{x\leq x_{i}} P_{i}$ | (eq. 1.1) |
| --- | --- | --- |

**B. Binomial distribution:** The success or failure cases in a random process are represented by a Bernoulli trial. Let the probability of the successful event be $p$and let $g(x)$be a new random variable resulting from transforming $X$with the function $g$. The expectation of $g(x)$, denoted $E\left[g(X)\right]$, is defined as:

|  | $E\left[g(X)\right]\leq\sum_{i}^{n} g(x_{i})P\left(X=x_{i}\right)$ | (eq. 2.1) |
| --- | --- | --- |

For the Bernoulli distribution, sample space can be represented with 0 for the failure case and 1 for the successful case. The probability of the failure is therefore $q=1-p$. This probability distribution can be described with equations below.

|  | $P\left(x\right)\equiv P\left(X=x\right)=p^{x}(1-p)^{1-x}$ | (eq. 2.2) |
| --- | --- | --- |
|  | $P\left(x\right)=\left\{\begin{matrix} 1-p=q, x=0 \\ p, x=1 \end{matrix}\right.$ | (eq. 2.3) |

The mean and variance are $\mu=p, \sigma^{2}=pq$.

**C. Poisson distribution:** The probability of homogeneous target cell encapsulation is similar to the Poisson process since the event is rare within the entire sampling space. In case of large $n$ and small $p$, $np$becomes a moderate size, e.g., more than one. This binomial distribution can be rewritten as Poisson distribution:

|  | $p\left(k\right)=P\left\{X=k\right\}=\frac{n!}{\left(n-k\right)!k!}\left(\frac{\lambda}{n}\right)^{k}\left(1-\frac{\lambda}{n}\right)^{n-k}\text{ where } \lambda=np$ | (eq. 3.1) |
| --- | --- | --- |

Based on the following large sample approximations, we get

|  | $\text{ }\left(1-\frac{\lambda}{n}\right)^{n}\approx e^{-\text{λ}},\text{ }\frac{n\left(n-1\right)\cdots(n-i+1)}{n^{k}}\approx1,\text{ and }\left(1-\frac{\lambda}{n}\right)^{\text{k}}\approx1$ | (eq. 3.2) |
| --- | --- | --- |

**D. System evaluation using CLT and Simple random sampling (SRS):** The sampling space reflects the entire sample conditions (i.e., population) due to large sampling number, since the overall process is random. For a random variable which has mean, $\mu$, and standard deviation $\text{σ}>0$, the central limit theorem (CLT) states that the sum of $n$ independent random variables has mean
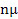
 and variance $n\sigma^{2}$ and show asymptotically Gaussian distribution. Equivalently, the random variable and statement can be described as:

|  | $X_{n}=\frac{X_{1}+\cdots+X_{1}-n\mu}{\sigma\sqrt{n}}=\sum_{i=1}^{n} \frac{X_{i}-n\mu}{\sigma\sqrt{n}} \text{ that }\lim_{n\to\infty} F_{X_{n}}\left(x\right)=\frac{1}{\sqrt{2\pi}}\int_{-\infty}^{x} e^{-t^{2}/2}dt$ | (eq. 4.1) |
| --- | --- | --- |

In particular the $X_{1}\cdots X_{n}$ are $n$ independent identically distributed random variables. Considering the sequence of $n$ mutually independent variables $X_{1}\ldots X_{n}$ with means $\mu_{k}$ and variances $\sigma_{k}^{2}$, the sum $S_{n}=X_{1}+\ldots+ X_{k}+\ldots+ X_{n}$ has mean and variance given by $\mu=\mu_{1}+\ldots+ \mu_{k}+\ldots+ \mu_{n}$ and $\sigma^{2}= \sigma_{1}^{2}+\ldots+\sigma_{k}^{2}+\ldots+\sigma_{n}^{2}$, respectively. The sequence follows the CLT if for every fixed $\alpha<\beta$, the following holds:

|  | $P\left(\alpha<\frac{S_{n}-\mu}{\sigma}<\beta\right)\to_{n\to\infty} \Phi\left(\beta\right)-\Phi\left(\alpha\right)$ | (eq. 4.2) |
| --- | --- | --- |

where $\Phi\left(t\right)$ stands for the distribution function of a standard normal distribution. A necessary and sufficient condition for this result to hold is that $X_{k}$ are mutually independent and uniformly bounded, i.e., $|X_{k}|<A$where $A$ is a positive constant. In practice, many phenomena can be considered as the result of the summation of many independent cases, yielding then, by the CLT, a distribution that closely resembles the normal distribution, for a moderate sample size [4]. Using CLT, based on sampling space of droplets can be used to estimate conditions of loading sample. These results were demonstrated under conditions of simple random sampling (SRS) as shown in **Table S3 and S4**.

**E. Model Assumptions**

**I-Modeling number of droplets that contain cells, $X_{d}$:**

$b_{n,p}(k)=P(X_{d}=k)=\left(\begin{matrix} n \\ k \end{matrix}\right)p^{k}q^{n-k}$, $0\leq k\leq n$, where, $\left(\begin{matrix} n \\ k \end{matrix}\right)=\frac{n!}{(n-k)!k!}$ (eq. 3.1 of main text)

**Assumptions:** In modeling number of droplets that contain cells, $X_{d}$, with a binomial distribution, a “trial” corresponds to a “droplet”, “success” corresponds to “ a droplet containing cells” and “failure” corresponds to “a droplet not containing cells”. The underlying assumptions for this model are: **(a)** there are n (i.e., fixed number of) trials (droplets) and the event of interest is the number of successes (i.e., number of droplets that contain cells in n droplets), **(b)** Each trial (i.e., droplet) has two outcomes: either droplet contains cells (success) or not (failure), **(c)** Probability of success is constant and equals to p and assumed to be fixed from droplet to droplet, and **(d)** Trials are independent in the sense that a droplet containing cells has no influence on another droplet’s containing cells and vice versa.

**II-Modeling number of cells in a droplet, $X_{c}$,**

$P(X_{c}=k)\approx e^{-\lambda}\frac{\lambda^{k}}{k!}$ (eq. 3.4 of main text)

**Assumptions:** In modeling number of cells in a droplet, $X_{c}$, with a Poisson distribution, an “event” is “the presence of a cell in a droplet”. The underlying assumptions for this model are: **(a)** the probability of observing a single event (i.e., cell) over a small space (droplet) is approximately proportional to the size of that droplet, **(b)** the probability of two events (cells) occurring in the same narrow (i.e., infinitesimally close) space is negligible, **(c)** the probability of a cell occurring within a certain space (or droplet) does not change over different spaces or droplets, and **(d)** The probability of a cell occurring in one space or droplet is independent of the probability of a cell occurring in any other non-overlapping space or other droplet.

**III-Modeling number of target cells in a droplet, $X_{t}$ with a Poisson distribution (see** (eq. 3.6 of main text))

**Assumptions:** In modeling number of target cells in a droplet, $X_{t}$, with a Poisson distribution, an “event” is “the presence of a target cell in a droplet”. The underlying assumptions for this model are as in II above with “cell” being replaced by “target cell”.

**IV-Modeling number of droplets that contain a single target cell, $X_{s}$ with** (eq. 3.6 of main text)

$=\frac{e^{-\lambda}\lambda^{k_{p}}}{k_{p}!}\times\frac{n!}{(n-k_{b})!k_{b}!}P^{k_{b}}(1-p)^{n-k_{b}}=\frac{\lambda n!}{e^{\lambda}(n-k_{b})!k_{b}!}P^{k_{b}}(1-p)^{n-k_{b}}$ (eq. 3.6 of main text)

**Assumptions:** In modeling number of droplets that contain a single target cell,$X_{s}$, conditional on $X_{d}>0$, it is assumed that $X_{d}$ and $X_{t}$ are independent, and hence PDF of $X_{s}$ can be written as the product of the two PDFs.

**References**

1. Moon S, Hasan SK, Song YS, Xu F, Keles HO, et al. (2010) Layer by layer three-dimensional tissue epitaxy by cell-laden hydrogel droplets. Tissue Eng Part C Methods 16: 157-166.

2. Sa JPMD (2007) Applied statistics using SPSS, STATISTICA, MATLAB and R. Berlin; New York: Springer.

3. Ross SM (2008) A first course in probability. Harlow: Pearson Education.

4. Grinstead CM, Snell JL (1997) Introduction to probability. Providence, RI: American Mathematical Society.
