# Supplementary figures and images for: Statistical Modeling of Single Target Cell Encapsulation
Source: PLoS One. 2011 Jul 21;6(7):e21580. doi: 10.1371/journal.pone.0021580 (PMC3140975; doi:10.1371/journal.pone.0021580)

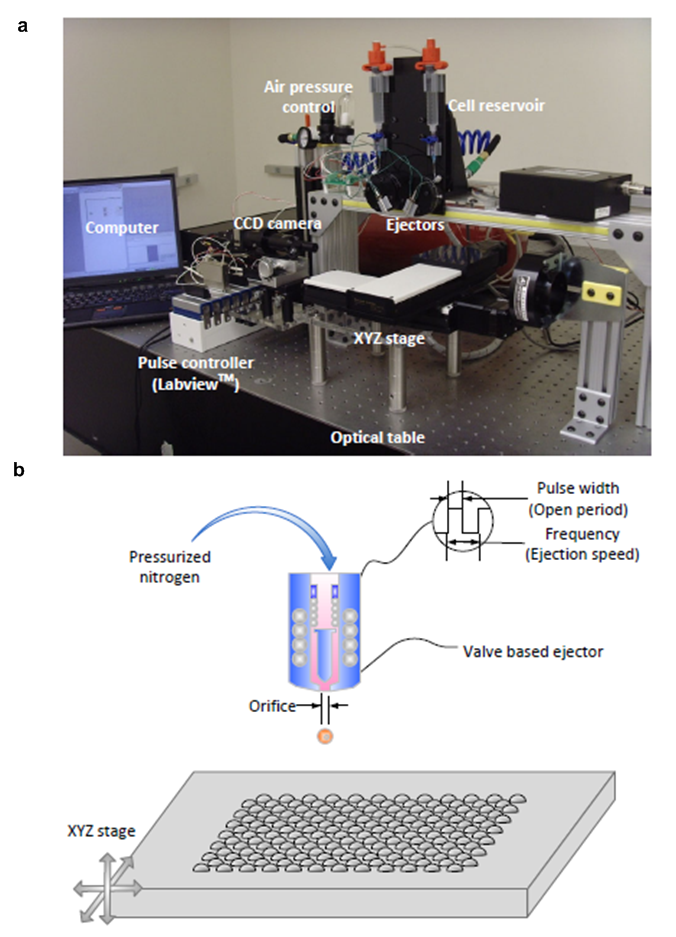

Supplement: Figure S1 — Image and illustration of a drop-on-demand cell encapsulation system. (a) Image of the setup. A computerized xyz stage was synchronized with a pulse controller, Labview™. The automated stage positioned the substrate with 5 µm spatial resolution. A 10× magnifying camera permitted in-situ imaging of the droplets. (b) Schematic of droplet ejector showed cells flowing into the valve driven by a controlled air pressure pulse. A heterogeneous sample, mixture of cells and media solution, was loaded into a reservoir. Each droplet was placed at a predetermined position (10×10 droplet array). (TIF) [file pone.0021580.s002.tif]
