# Supplementary material for: Statistical Modeling of Single Target Cell Encapsulation
Source: PLoS One. 2011 Jul 21;6(7):e21580. doi: 10.1371/journal.pone.0021580 (PMC3140975; doi:10.1371/journal.pone.0021580)
Supplement: Table S1 — Abbreviations and descriptions for statistical modeling of single target cell encapsulation. (DOC) [file pone.0021580.s003.doc]

**Table S1.**

| Abbreviations | Descriptions |
| --- | --- |
| *E* | Set of all events (i.e., universe) |
| *A* | Set of events |
| *P* | Probability of an event |
| *k* | Number of successes |
| *n* | Sampling number (i.e., droplet number or sample size) |
| *ε* | Tolerance |
| *1-α* | Confidence level |
| *λ* | Poisson distribution parameter |
| *μ* | Mean |
| *σ****^2^*** | Variance |
| *PDF* | Probability distribution function |
| *CLT* | Central limit theorem |
| *SRS* | Simple random sampling |
| *LLN* | law of large numbers |
