# Supplementary material for: Statistical Modeling of Single Target Cell Encapsulation
Source: PLoS One. 2011 Jul 21;6(7):e21580. doi: 10.1371/journal.pone.0021580 (PMC3140975; doi:10.1371/journal.pone.0021580)
Supplement: Table S2 — Random variables in our data set and representative meanings of each variable for random cell encapsulation process. Three different random variables (i.e., X d, X c, and X t) were defined in discrete independent domain and one dependent variable (i.e., X s) was defined by a combination of independent variables for overall process efficiency. Three variables were used to represent percentage of empty droplets, effect of number of cells in droplets as a function of loading cell concentrations, and target cell concentrations, respectively. (DOC) [file pone.0021580.s004.doc]

**Table S2.**

| **Data set** | **Variable** | **Value domain** | **Type** | **Representative meaning** |
| --- | --- | --- | --- | --- |
| Number of droplets that contain cells | *X*_d_ | { 0, 1, …, *k* } | Discrete independent | % of empty droplets |
| Number of cells in a droplet | *X*_c_ | { 0, 1, …, *l* } | Discrete independent | effect of loading cell concentrations |
| Number of target cells in a droplet | *X*_t_ | { 0, 1, …, *m* } | Discrete independent | effect of target cell concentrations |
| Number of droplets that contain a single target cell | *X*_s_ | { 0, 1, …, *n* } | Discrete dependent | overall process efficiency |
