# Supplementary material for: Statistical Modeling of Single Target Cell Encapsulation
Source: PLoS One. 2011 Jul 21;6(7):e21580. doi: 10.1371/journal.pone.0021580 (PMC3140975; doi:10.1371/journal.pone.0021580)
Supplement: Table S3 — Values of Poisson coefficient, λ, for four different percentage of target cell mixture and four different cell loading concentrations at reservoir. Probability of encapsulating a single target cell in a droplet was presented by probability distribution function, P(X s). The number of homogeneous droplets was modeled using Poisson distribution in a random variable space, i.e., number of target cells. The model was verified using a coefficient, λ, and experimental results. Average number of cells, λ, for four different cell loading concentrations and target cell concentrations were determined. As cell loading density increases, target cell concentration, λ values increase, from λmin = 0.03 to λmax = 0.95. Based on these experimental and analysis results, statistical models can be determined based on λ values, e.g., λ = 0.10- for 1.0×10^5^ cells/ml with 10% target cell mixture. (DOC) [file pone.0021580.s005.doc]

**Table S3.**

| **% target cell mixture** | **Cell loading concentrations at reservoir** | | | |
| --- | --- | --- | --- | --- |
|  | **0.5 × 10^5^ cells/ml** | **1.0 × 10^5^ cells/ml** | **1.5 × 10^5^ cells/ml** | **2.0 × 10^5^ cells/ml** |
| 10% | 0.03 | 0.10 | 0.16 | 0.28 |
| 20% | 0.05 | 0.16 | 0.34 | 0.36 |
| 30% | 0.08 | 0.28 | 0.60 | 0.74 |
| 50% | 0.16 | 0.68 | 0.75 | 0.95 |
