# Supplementary material for: Statistical Modeling of Single Target Cell Encapsulation
Source: PLoS One. 2011 Jul 21;6(7):e21580. doi: 10.1371/journal.pone.0021580 (PMC3140975; doi:10.1371/journal.pone.0021580)
Supplement: Table S4 — Statistical modeling results for drop-on-demand single target cell encapsulation. (a) Randomness of process was verified by three variables, number of samples (n), tolerance (ε), and confidence level (1−α) using an inequality(**). Following the law of large numbers (LLN), minimum sample number was determined as 100 droplets for 90.0% confidence level and 15.0% tolerance. This sampling volume of a droplet represented (0.76 µl = 10×10×7.6 nl) 0.76% of the total volume of the ejection reservoir (0.1 mL). (b) Random processes have different PDFs in accordance with their parameters, i.e., droplets that contain cells (Xd), number of cells in a droplet (Xc), number of target cells per droplet (Xt), and droplets that contain single target cells (Xs). These four random variables are represented by binomial, Poisson, and normal distributions to statistically model the cell encapsulation process. We investigated probability values and parameters, λ, for each case with respect to the cell loading concentration, cell volume fraction, and percentage of target cells. (c) In the case of SRS process, statistical characteristics of a random sampling volume can represent the characteristics of a large population, and the required number of droplets (i.e., sample size) is based on the CLT. In our experiments, the target cell fraction (F%) shows same concentration as the reservoir concentration for 10.0% to 50.0% at 1.0×10^5^ cells/ml concentration (Copt) under conditions of 90.0% confidence level and 15.0% tolerance. (DOC) [file pone.0021580.s006.doc]

**Table S4.**

|  | **Process description** | **Variable** | **PDF^(1)^** | **Results** |
| --- | --- | --- | --- | --- |
| (a) | Randomness  (cell encapsulation) | *n, ε, α* | Based on  normal distribution  (LLN) | - number of samples: 10 × 10  - sampling volume fraction^(2)^: 0.76%  - confidence level: 90.0%  - tolerance: 15.0% |
| (b) | Droplets that contain cell(s)  (cell encapsulation) | *X_d_* | Binomial distribution | *^(*)^*$f\left(X_{d}=1\right)\in(0.\text{27,0.87)}$  - cell loading concentration  - cell volume fraction^(3)^ |
|  | Cells in a droplet  (cell distribution) | *X_c_* | Binomial distribution,  Poisson distribution | $f\left(X_{c}=\lambda\right)\in\text{ (}0.\text{4,2}.\text{2)}$ - cell loading concentration |
|  | Target cells in a droplet  (target cell distribution) | *X_t_* | Poisson distribution | $f\left(X_{t}=\lambda\right)\in\text{(}0.0\text{3,}0.\text{95)}$ - cell loading concentration  - percentage of target cells |
|  | Droplets that contain  single target cell  (target droplet distribution) | *X_s_* | Poisson distribution | $f\left(X_{s}=\text{ }\lambda\right)\in(0.0\text{3,}0.\text{95)},\text{ P}\in\text{(0.27,0.87)}$ - cell concentration  - percentage of target cells |
| (c) | CLT for SRS^(4)^  (target droplet selection) | *ε, α, C_opt_, F%* | Based on  normal distribution  (CLT for SRS) | - confidence level: 90%  - tolerance: 15%  - optimum cell concentration: 1.0 × 10^5^  - target cell fraction^(5)^: 10 % to 50% |

^(*)^ *for example f(X_d_=1) = probability of droplet containing cells ranges from 27% to 87%*

^(**)^ Sampling number (i.e., number of droplets): n ≥ pq/(ε^2^α), where, p = 0.6, q = 0.4

^(1)^PDF: Probability Distribution Function

^(2)^Sampling volume fraction (%) = total sample set volume / entire volume (100 µl) × 100 = 0.76%

^(3)^Cell volume fraction = number of cells × single cell volume / droplet volume, assuming an average cell size of 10µm.

^(4)^CLT (Central Limit Theorem) and SRS (Simple Random Sampling)

^(5)^Target cell fraction = number of target cells / number of whole cells
